# Prevalence and determinants of diarrhoea among infants in selected primary health centres in Kaduna north local government area, Nigeria

**DOI:** 10.11604/pamj.2017.28.109.8152

**Published:** 2017-10-04

**Authors:** Magbagbeola David Dairo, Tosin Faisal Ibrahim, Adetokunbo Taophic Salawu

**Affiliations:** 1 Department of Epidemiology and Medical Statistics, Faculty of Public Health, College of Medicine, University of Ibadan, Nigeria; 2 Nigeria Field Epidemiology and Laboratory Training Programme, Abuja, Nigeria

**Keywords:** Exclusive breastfeeding, infant diarrhoea, immunization status, infant care, maternal health education

## Abstract

**Introduction:**

Despite efforts toward the prevention and management of diarrhoea, associated mortality among infants has remained high in Northern Nigeria. This study was designed to determine the prevalence and identify determinants of diarrhoea among infants in Kaduna North Local Government Area (KNLGA), Nigeria.

**Methods:**

In a cross-sectional survey 630 mothers of infants attending three primary health care centers were interviewed. Data was collected on socio-demo graphic characteristics, infant care practices, infant diarrhoea history and mothers knowledge of causes, symptoms and management of diarrhea. Data were analyzed using descriptive statistics, Chi-square, and logistic regression tests at 5% level of significance.

**Results:**

Mothers' mean age was 27±5.5 years and 46.1% had secondary education. Infants' mean age was 22.4± 12.8 weeks and 50% were females. Prevalence of diarrhoea in the two weeks preceding the study was 21.1%. Only 11.7% of mothers had poor knowledge of diarrhoea. About 76.3% of mothers always washed their hands with soap after cleaning infants' perineum. Majority of infants (84.6%) completed age appropriate immunization while 31.6% were exclusively breastfed. Infants whose mothers sometimes (OR=2.32; 95% CI: 1.4-3.87) or never washed (OR=2.64; 95% CI: 1.19-5.82) their hands with soap after cleaning the infants perineumand those with incomplete age appropriate immunization (OR=1.87, 95% CI: 1.2-2.896) were more likely to have diarrhoea.

**Conclusion:**

Promotion of hygiene and nutrition education for mothers particularly on proper infant feeding practices, hand washing practices and complete immunization of infants is needed to address the diarrhea determinants.

## Introduction

Diarrhoea is defined as passage of loose or watery stools at least three times per day, or more frequently than normal for an individual. Frequent passing of formed stools is not diarrhoea [1]. Babies fed only breast milk often pass loose, “pasty” stools; this also is not diarrhoea. Mothers can usually determine when their children have diarrhoea [2]. Each year, an estimated 2.5 billion cases of diarrhoea occur among children under five years of age, and estimates suggest that overall incidence has remained relatively stable over the past two decades, more than half of these cases are in Africa and South Asia, where bouts of diarrhoea are more likely to result in death or other severe outcomes [3, 4]. Diarrhoea due to infection is widespread throughout the developing world. According to WHO, in Southeast Asia and Africa, diarrhoea is responsible for as much as 8.5% and 7.7% of all deaths respectively. In Africa, it has been estimated that every child has five episodes of diarrhoea per year and that 800,000 children die each year from diarrhoea and dehydration. Diarrhoea is the second biggest killer of children in Nigeria, responsible for about 16% of child's death every year. Nigeria was estimated to have a total number of annual child deaths due to diarrhoea to be 151,700 (WHO, 2009). Diarrhoea was the most commonly reported cases of waterborne infection in the three towns in North Western Nigeria. In Nigeria, contaminations of drinking water with pathogens have also been reported in several towns [4, 5]. The infectious agents that cause diarrhoea are present or are sporadically introduced throughout the world. Although, diarrhoea is a rare occurrence for most people who live in developed countries where sanitation is widely available, access to safe water is high, personal and domestic hygiene is relatively good. Diarrhoea due to infection continues to be widespread throughout the developing world. Incidence of diarrhoea is highest in the first two years of life and declines as a child grows older. Diarrhoeal disease mostly affects children under two years of age, and may be life-threatening, particularly in those who are malnourished or have impaired immunity [6, 7]. These causative pathogens of diarrhoea are found in faecal matter and are transmitted from the stool of one individual to the mouth of another (faecal-oral transmission ) which may be spread through contaminated water, food, hands, eating and drinking utensils, flies, and dirt under fingernails [8]. Apart from causing significant mortality and morbidity, diarrhoea is also a leading cause of malnutrition in children under-five years old due to its association with poor nutrient absorption and appetite loss. Frequent bouts of acute diarrhoea seriously debilitate children. With each successive episode, a child moves further away from his or her normal weight for age thereby greatly increasing the risk of malnutrition and impaired child development.

Diarrhoea is an immediate health threat to children; it also has long-term negative effects on the country socio-economic development [9]. Infant diarrhoeal infections have sequelae which impact on children lives. Several studies have quantified these long-term effects of early childhood diarrhoea and parasitic infections during the critical, formative first two years of life. The long-term effects include growth shortfalls, substantially impaired physical fitness, diminished cognitive capacity and delayed achievement at school [10]. Diarrhoea indirectly, has adverse effect on health, educational performance and school attendance of these children. Thus it can be stated that diarrhoea affects key areas of development such as heath, economy and education. In Nigeria, there is a regional discrepancy in the distribution of diarrhoea. There is an increasing prevalence of diarrhoeal disease among infants in the northern part of Nigeria than in the south [11]. However, there is a dearth of information on factors associated with the increasing occurrences of the disease among under-five children in Northern Nigeria. Factors associated with this increase needs to be further investigated. Literature from different countries usually has distinct factors associated with the occurrence of diarrhoea. For effective control and prevention of diarrhoea there is need for information on factors related to increased risk of diarrhoeal disease. Thus information from this study will be vital to health authorities in the study area in terms of better management of the disease. The results of the study can also be used to guide the formulation of policies relating to diarrhoea management in the study area. Thus, this study determined prevalence and determinants of diarrhoea among infants in Kaduna North Local Government Area, Nigeria.

## Methods

**Study design, area and population:** A hospital based cross sectional study was conducted in KNLGA between May, 2011 and July, 2011. Zakariya Memorial Center, PHC Badarawa, and PHC Unguwar Shanu were the site for the study. These Primary Health Care Centres (PHCs) on immunization days served a large number of children (an average of 60 children per facility). The study population were mothers/caregivers and their infants between the ages of one month and 12 months within the study site, that provided written or verbal informed consent.

**Sample size and sampling procedure:** Leslie Kish formula was used to calculate the sample size, based on an assumption that 27% of two-week prevalence of diarrhea in Jos Nigeria. The calculated total sample size was 630 mother-infant pair. A three-staged sampling procedure was employed, first; three wards were selected from the 11 wards in the selected Local Government Area (Kaduna North LGA) by simple random technique. One PHC was selected at random from each of the three randomly selected wards by balloting. All mothers of eligible infants in each of the selected PHCs were recruited into the study till the sample size of 630 was attained.

**Data collection methods and quality:** A pretested semi-structured interviewer-administered questionnaire was used to collect data on socio-demographic characteristics, infant care practices, diarrhoea history of infants and knowledge of causes, symptoms and management of diarrhea.

**Data analysis:** The data were inputted into SPSS. Questionnaires and data collected were safely kept. Analysis was done using SPSS version 21.0. Data cleaning was done after all the data had been entered. Descriptive statistics and inferential statistics (chi-square and logistic regression) were used for the data analysis. In all analyses, P<0.05 were considered as a significant.

**Ethical considerations:** Ethical approval for the study was obtained from UUUCH Ethics Committee. Permission was obtained from officer in-charge of each PHCs before data were collected at the various immunization centres. Consent forms and questionnaires were translated, back translated and administered in respondents' native languages (Hausa) for respondents who do not speak English Language. Consent was obtained from the participants after explaining the study and showing full understanding of the study (see appendix for consent form). Completed forms were kept in sec.8ured setting where no other persons could have access to the information obtained from respondents. All information was used for the purpose of the research only.

## Results

**Socio-demographic characteristics of mothers:** The sample consisted of 630 mothers-infant pair, the ages of the mothers ranged from 12 to 46 years with mean of 27.8 s±5.5 years. Majority (98.6%) of the respondents were married. The main religion of the respondents was Islam (57.9%). The main ethnic groups were Hausa (42.4%), although there are other minority ethnic groups (31.6%). Almost half of the mothers (46.6%) had secondary education while only 10.2% had no formal education (Table 1).

**Table 1:** Socio-demographic characteristics of mothers/caregiver and their infants in selected PHCs in Kaduna North local government area

Characteristics		N=630	Percentage
**Mothers age (in years)**	10-29	30	4.8
	20-29	360	57.1
	30-39	226	35.9
	40-49	14	2.2
**Mothers Religion**	Christianity	265	42.1
	Islam	365	57.9
**Mothers Ethnicity**	Hausa	267	42.4
	Yoruba	105	16.6
	Igbo	59	9.4
	Other minority	196	31.6
**Mothers Education**	No formal Education	64	10.2
	Primary Education	80	12.7
	Secondary Education	294	46.6
	Tertiary Education	192	30.5
**Sex of the Infants**	Male	318	50.5
	Female	312	49.5
**Infants’ age (in weeks)**	>8	156	25
	9-16	111	18
	17-24	139	22
	25-32	21	3
	33-40	164	26
	41-44	39	6
**Infants’ Birth order**	First	215	34.1
	Second	151	24.0
	Third	115	18.2
	Fourth to Sixth	135	21.5
	Seventh to eleventh	14	2.2

++ Mean age of infants=22.4± 12.8 weeks

**Socio-demographic characteristics of infants:** The age of the infants ranges from 1to11 months with mean 5.6±3.2 months and 50.5% were females. The birth order of the infants ranged from 1 to 11 (Table 1).

**Infant care practices of mothers/care giver:** Table 2 Shows child care practices of the respondents. Most (85.9%) of the respondents always wash their hands with soap after using the toilet. A reasonable proportion (76.3%) of the respondents washes their hands always with soap after cleaning infant's perineum. A high percentage (84.6%) of the infants had completed immunization status for their age.

**Table 2:** Infant care practices of mothers/caregiver

Infant care practices		Frequency	Percentage
**Mothers wash hands with soap after using the toilet (N=630)**	Always	541	85.9
	Sometimes	83	13.1
	Never	6	1.0
**Mothers wash hands after cleaning infants perineum (N=630)**	Always	481	76.3
	Sometimes	119	18.9
	Never	30	4.8
**Infants immunization status (N=630)**	Complete for age	533	84.6
	Incomplete for age	97	15.4
**Ever breastfed infant (N=630)**	Yes	630	(100)
**Currently breast feeding infant (N=630)**	Yes	629	99.8
	No	1	0.2
**Type of breast feeding practice (N=630)**	Exclusive	199	31.6
	Predominant	192	30.5
	Partial	239	37.9
**Pre-lacteal feeding (N=274)**	Water	159	58.0
	Honey	44	16.1
	Glucose water	42	15.3
	Zam-zam and date	29	10.6
**Reasons for pre-lacteal feeding (N=274)**	Infant’s mother is sick	21	7.7
	Makes infants healthy	64	23.4
	Reduce thirst	10	3.6
	Cleans infants stomach	8	2.9
	Tradition	93	33.9
	No enough breast milk	69	25.2
	Others	9	3.3
**Infants that did not receive colostrums (N=16)**	Infant formula only	12	75.0
	Others	4	25.0

**Infant feeding practices of mothers/care giver:** Table 2 Shows also the various feeding practices of the mothers among the infants surveyed. About 99.0% of infants were still breast feeding at the time data was collected. Initiation of breastfeeding commenced in most (82.9%) of the infants within 12 hours of delivery. Only 31.6% of the infants were exclusively breast fed and 11.3% of EBF infants were fed with breast milk only for six months as at the time of interview. In total 30.5% infants were predominantly and 37.9% of the infants were partially breast fed. Less than half (43.5%) of the six hundred and thirty infants were pre-lacteally fed with honey (16.1%), glucose water(15.3%), combination of zam-zam (holy water from Saudi Arabia) and date fruit (10.6%) after birth. Culture/ traditional practice (33.9%) were the reason why mothers pre-lacteally fed their infants' and 62.2% of infants were given breast milk within three days of birth.

**Diarrhoea experience of infants:** More than half (57.5%) of the infants had ever experienced diarrhoea at one time or the other in their life while the two weeks period prevalence of diarrhoea was 21.1%. There were more Infants below 6 months who had diarrhoea experience (71.7%) compared to infants above six months (28.3%). Most (43.4%) of the infants diarrhoea experience began at age 3 and 4 months. Most infants with diarrhoea (78.4%) were treated at the hospital while only 16.7% were treated at home.

**Knowledge of diarrhoea of mothers:** The mean knowledge score was 7.6±1.0 out of 9 points, with many (88.3%) of the respondent scoring above 7 points. These respondents were classified as having a good knowledge on diarrhoea.

**Determinants of infant diarrhoea: multivariable result**

**Socio demographic determinants:** Mothers'/ care givers' educational qualification [OR 1.6 95% CI =1.1-2.4] was significantly associated with prevalence of infant diarrhea in bivariate analysis of socio-demographic variables (Table 3).

**Table 3:** Summary of logistic regression analysis of determinants of infant diarrhoea

Variable		Odds Ratio	95% confidence interval		P value
			Lower	Upper	
**Education**	Secondary/Tertiary	1.00			
	No formal/primary	1.6	1.1	2.4	<0.01
**Wash hands after using the toilet.**	Always	1.00			
	Sometimes	1.50	0.841	2.691	0.169
**Wash hands after cleaning infant’s perineum**	Always(reference)	1.00			
	Sometimes	2.32	1.40	3.87	0.016*
	Never	2.64	1.19	5.82	0.001
**Immunization Status for Infants age**	Complete for age (reference)	1			
	Incomplete for age	1.87	1.210	2.896	0.05
**Infant’s commencement of complementary food**	1 month	2.18	0.56	8.46	0.26
	2 months	2.82	1.02	7.79	0.05
	3 months	1.99	0.98	4.18	0.07
	4 months	2.88	1.33	6.20	0.007
	5 months	2.53	1.26	5.06	0.009
**Feeding practice**	Exclusive (reference)	1.00			
	Predominant	2.78	1.79	4.29	0.001
	Partial	4.59	3.20	6.99	0.001
**Duration of breast feeding**	Less than six month	1.00			
	For six months	0.4	0.20	0.7	0.001
**Knowledge**	Good Knowledge	1.00			
	Poor Knowledge	3.3	1.9	5.7	0.001

**Infant care practice determinants:** Diarrhoea was more common among infants whose mothers sometimes (OR =2.32; CI: 1.40-3.87) and never (OR= 2.64; CI: 1.19-5.82) wash their hands with soap after cleaning infants perineum, and infant's whose immunization status was not up to date (OR= 1.87; CI: 1.210-2.896). Diarrhoea was also commoner among predominantly (OR= 2.78, CI: 1.79 - 4.29) and partially breast fed infants (OR= 4.59, CI: 3.20- 6.99) than exclusive breast fed infants. Complementary feeding at two months (OR=2.82; CI=1.02- 7.79), four months (OR=2.9, CI=1.33- 6.20) and five months (OR=2.53, CI=1.26-5.06) were significantly associated with diarrhoea among infants. **Diarrhoea is also significantly associated with infants whose Mothers/care giver had poor knowledge of causes of diarrhoea (OR=3.3, CI = 1.9 ' 5.7) (**Table 3**).**

## Discussion

The two weeks period prevalence of diarrhoea was 21.1%. However, the prevalence of diarrhoea in this study was lower than the prevalence of diarrhoea in Jos North central Nigeria which is 27% [12], but higher than the prevalence in in Ondo state, a predominantly agrarian state in the high rainfall region of south western Nigeria where the prevalence of diarrhoea was 8.1%, [12] further confirming regional variation in the prevalence of diarrhoea [13]. The observation that mothers with at most primary education had higher probability of having diarrhoea than those with at least secondary education corroborate findings of a study conducted in Guinea Bissau in which education was significantly associated maternal morbidity [13]. The result from the logistic regression analysis of predictors of diarrhoea shows significant association with washing hands with soap after cleaning infants' perineum. Infants whose mother sometimes and never wash their hands with soap after cleaning infants perineum were more likely to have diarrhoea than infants whose mother always wash their hands with soap after cleaning their infants perineum (OR =2.32; 95% CI: 1.40 -3.87) and (OR=2.64; 95% CI: 1.19- 5.82) respectively. Findings from this study corroborate with finding of Curtis and Cairncross that there are clear scientific evidence that support the practice of hand washing, stating that hand washing reduces diarrhea risk by 47% [14]. According to the WHO, Immunizations help reduce deaths from diarrhoea in two ways: by directly preventing infections that cause diarrhoea (such as rotavirus) and by preventing infections that can lead to diarrhoea as a complication of an illness (such as measles). The impact of immunization status on the prevalence of diarrhoea is corroborated by findings from this study. In this study infants whose immunization status was not up to date for their age were found more likely to experience diarrhoea than infants whose immunization status was up to date for age. The WHO recommends exclusive breast feeding for the first six months of life and continued breastfeeding until the age of two years. Predominantly and partially breast fed infants were significantly more likely to have reported diarrhoea than exclusively breast fed infants Similarly, in this study, infants who started on complementary foods at any period between two and six months were significantly more likely to have reported diarrhoea than infants who had never being fed with complementary foods. The observation that mothers knowledge of diarrhoea symptoms, causes and prevention of diarrhoea was associated with lower prevalence of diarrhoea among their infants are in agreement with findings of a study conducted in Ibadan in which knowledge was significantly associated practice of child survival strategy [15].

## Conclusion

The findings of this study demonstrated that diarrhoea is a common experience among infants in KNLGA and that a large proportion of infants have experienced diarrhoea in the last two weeks. The findings from the study also show that the factors associated with the occurrence of diarrhoea among infants in KNLGA were modifiable maternal factors (poor maternal education, partial breast feeding, and predominant breast feeding and poor maternal knowledge of diarrrhoea). It is therefore strongly recommended that diarrhoea prevention programmes should focus on these modifiable factors.

### What is known about this topic

- Previous reports have documented the influence of nutrition, immunization against certain childhood killer diseases and infant weaning diet on the prevalence of diarrhoea among infants;
- The findings reported in this study corroborates the earlier documented facts in the literature;
- However the role of maternal factors in the aetiology of infant diarrhoea in unclear in documented literature.

### What this study adds

- This study highlights the role of maternal hygiene factors on the prevalence of diarrhoea among infants in the community. The study provides evidence that maternal hygiene is a critical factor in the sustenance of the cycles of episode of diarrhoea among infants in this community;
- The findings from this study suggests that control of diarrhoea among infants will benefit greatly from intervention at maternal level with particular focus onhand-washing before feeding infants and also after cleaning up infants.

## Competing interests

Author's declare no competing interest.
